# Maternal perspectives on the use of probiotics in infants: a cross-sectional survey

**DOI:** 10.1186/1472-6882-14-366

**Published:** 2014-09-29

**Authors:** Sarah L Bridgman, Meghan B Azad, Catherine J Field, Nicole Letourneau, David W Johnston, Bonnie J Kaplan, Anita L Kozyrskyj

**Affiliations:** Department of Pediatrics, University of Alberta, Edmonton, Canada; Department of Agriculture, Food & Nutritional Science, University of Alberta, Edmonton, Canada; Faculties of Nursing & Medicine (Pediatrics), Calgary, Canada; Department of Pediatrics, Alberta Children’s Hospital, Calgary, Canada

**Keywords:** Probiotics, Infant, Mother, Nutrition, Natural health products, Cross-sectional survey

## Abstract

**Background:**

Probiotic products that may modify the intestinal microbiota are becoming increasingly available and known to consumers due to their potential to prevent or treat many pediatric health conditions. As scientific knowledge of the health benefits of probiotics increases, it is important to identify factors that may prevent their successful integration into patient care as well as to ensure effective translation of research findings. The aim of this study was to describe maternal perspectives on probiotics and their use in infants.

**Methods:**

Mothers with a child aged two years or younger enrolled in the Alberta Pregnancy Outcomes and Nutrition (APrON) study were invited by email to complete a 29 item self-administered web-based questionnaire.

**Results:**

A total of 413 mothers of the 1327 contacted completed the questionnaire. The majority (99.3%) of respondents had heard of probiotics and were aware that they contained live bacteria (87.0%); 89.3% had used a product containing probiotics themselves but only 50.8% had given one to their infant. Most mothers indicated they believed that probiotics were beneficial (73.1%) and none thought they were harmful. Over a third of mothers did not feel informed enough to make a decision on whether probiotics were safe to use in infants (36.6%).

**Conclusions:**

The study demonstrates that awareness and understanding of probiotics is high among mothers in Alberta, Canada. However, there is still uncertainty regarding the benefit of probiotics as well as safety in infants which could be important factors determining therapeutic use in the future. Further studies that demonstrate beneficial effects and safety of probiotics in healthy infants as well as targeted knowledge translation should help to address these potential concerns.

## Background

Due to the association between the intestinal microbiota and human health [1–3], attention has focused on products that can beneficially manipulate the microbiota profile. Probiotics are commonly defined as live microorganisms, which when administered in adequate amounts confer a health benefit on the host [4]. To date, a large number of studies have been conducted to assess the potential beneficial effect of probiotics in the treatment and prevention of pediatric disease, including acute gastroenteritis, antibiotic associated diarrhea, necrotizing enterocolitis (NEC), constipation, allergic disease, colic and respiratory infections [5]. A recent review of the evidence by the Canadian Paediatric Society states that physicians should consider the use of probiotics in infants for the prevention of antibiotic-associated diarrhea, treatment of acute diarrhea, in the prevention of NEC in preterm infants and to decrease symptoms of colic and irritable bowel syndrome [6]. In contrast, other reviews have concluded that while a number of randomized controlled trials demonstrate encouraging results, clear clinical benefits of probiotics for prevention of disease in infants is not yet convincing [5, 7, 8].

Efficacy of probiotics depends on the species and strain of bacteria, as well as the dosage and length of administration. Heterogeneity in these variables among studies as well as inadequacies in study design has likely contributed to the conflicting outcomes in probiotic studies to date, hampering their translation into evidence-based recommendations [9, 10]. In addition, several concerns exist regarding the use of probiotics in infants due to their immature gastrointestinal and immune systems. Although extremely rare, probiotic bacteria have the potential to translocate through the gut epithelium and cause bacteraemia or sepsis [11]. The long term effect of administration of bacterial strains during the early development of the gut microbiota is also unknown. A recent study evaluating the safety of probiotics used in young children aged 0 – 2 years found no major safety concerns but highlighted that inconsistent and incomplete reporting of adverse events in studies and variation of strains and dosage used limit the generalizability of conclusions [12]. Overall, although predominantly deemed safe for use in healthy term infants, there is a consensus that more strain-specific safety data in this population group is needed, as well as evidence on longer term effects [5, 8, 12, 13].

In many countries, probiotics are categorized as foods or dietary supplements rather than medicines and therefore can be readily purchased in grocery and health food stores without a prescription or professional advice. There is growing availability of probiotic products marketed for infants and young children which include yogurts, liquid supplements and weaning foods. Probiotics are also now included in a number of infant formulae worldwide as evidence of the presence of live bacteria in breast milk increases [14].

Given the recent scientific attention, coupled with the increasing availability of probiotic products, it is important that practitioners have an understanding of patient knowledge and opinions surrounding probiotics, as well as their current use in infants to enable effective translation of research knowledge as it becomes available. The aim of this cross-sectional survey of mothers was to ascertain extent of probiotic use in infants, and maternal knowledge and opinions on their safety and effectiveness.

## Methods

### Study subjects

Mothers were recruited via email from the Alberta Pregnancy Outcomes and Nutrition (APrON) cohort study. APrON is an ongoing prospective general birth cohort of pregnant women and their infants from Calgary and Edmonton, Alberta (Western Canada) [15]. In Calgary, women were enrolled in APrON primarily through prenatal clinics, although community advertising and media coverage were also used. Due to differences in obstetrical care between the cities, women in Edmonton were recruited primarily through local media coverage, paid advertising and recruitment tables at shopping malls, recreational centres and community fairs. An APrON webpage was also used to recruit participants in both cities. After women expressed interest in the study, APrON research assistants contacted prospective participants to describe the study in more detail. Pregnant women over the age of 16 years with a gestation <27 weeks, and living in or near Calgary or Edmonton were eligible. Women were excluded if they were unable to answer questions in English or if they planned to move out of the region during the timeframe of the study. Mothers in APrON are self-reported to be primary caregivers of their infants. For the current survey, mothers were eligible for inclusion if they had a child aged two years or younger enrolled in APrON at the time of the survey administration and had given consent to be contacted about other studies outside of APrON.

### Survey

The self-administered web-based survey consisted of 29 questions in themed sections. We report here on responses to 9 questions relating to maternal awareness of probiotics, use in self and infants, as well as opinions surrounding probiotic use in infants. Standard demographic information was also captured as part of the survey. The survey was constructed and validated by six researchers in child health with expertise in microbiota research, nutrition, psychology, ethics and questionnaire design. It was subsequently piloted on 5 pregnant women or new mothers to verify understanding; pilot testing resulted in minor changes to the wording of some questions. The survey was conducted using Checkbox® v4.7, hosted on a secure server at the University of Alberta and was completed anonymously. Mothers who completed the survey were entered into a lottery prize draw to win a gift card. An email reminder was sent approximately 3 weeks after the initial invitation to encourage participation.

### Ethical considerations

The research protocol and all study materials and incentives were approved by the Health Research Ethics Board of the University of Alberta and the Cojoint Health Research Ethics Board at the University of Calgary. Consent was obtained from all participants before completion of the survey.

### Data analysis

Frequencies were compiled for all variables. Chi-square analysis was used to determine the association between use of probiotics in infants, sociodemographic characteristics and maternal opinions of probiotic benefit and safety. Mean differences in maternal opinion on safety across groups of commonly used products in infancy, including probiotics, was tested using the non-parametric version of repeated-measures analysis of variance (Friedman Test) with post hoc Wilcoxon signed-rank tests to further explore differences in perceived safety between probiotics specifically and all other food/products. The Wilcoxon signed rank test was used to explore differences in the intended use of probiotics based on a number of hypothetical scenarios of health effects compared to intention to use if no health effects were demonstrated. Responses to open questions were hand coded into relevant themes to enable reporting of frequencies. Statistical analysis was performed using IBM SPSS Statistics for Windows version 21.0

## Results

During April 2012, 1327 women who had enrolled in the APrON cohort study between September 2009 and December 2011 were invited by email to participate in the survey. Of the 1327 contacted, 426 subjects followed the link to the survey consent and information page. In the final analysis, 13 subjects were excluded (two did not give consent; 11 gave consent but did not complete any of the questions). In total, 413 mothers completed the survey after giving consent, representing a response rate of 31%.

The characteristics of the mothers are presented in Table 1. The majority were 25 years of age or older (98.5%), married (86.9%), had completed a university degree or higher (77.9%), had a total household income of over $70,000 (83.5%), were of European origin (74.7%) and were born in Canada (82.2%). They were representative of the APrON cohort in age, household income, marital status and place of birth; however, our sample contained a larger proportion of more highly educated mothers (93.1 vs 88.3% respectively held a trade, technical or higher degree). The mean age of the youngest child in the household at the time the survey was completed was 11.8 months.

**Table 1:** **Characteristics of survey participants**

Demographic variables	%
*Age range (N = 388)*	
18-24	1.5
25-34	66.0
35-49	32.5
*Marital Status (N = 389)*	
Married	86.9
Common-law/Living with partner	10.0
Single/Separated/Divorced/Widowed	3.1
*Highest Level of Education (N = 389)*	
Less than high school	0.3
High school	6.7
Technical/vocational/trade	15.2
University undergraduate	47.3
University post-graduate	30.6
*Household Income (N = 382)*	
< $20,000	1.8
$20,000-$39,999	2.6
$40,000-$69,999	12.0
$70,000-$99,999	26.4
$100,000 or more	57.1
*Children (under 18 yrs) in the Household ( N = 389 mean ± SD)*	1.6 (±0.72)
*Age of youngest child in months (N = 388 mean ± SD)*	11.8 (±6.59)
*Ethnic origin (N = 383)*	
First Nations/ Metis	1.3
European	74.7
Caribbean	0.3
Latin/central/south American	2.3
African	1.0
Arab	0.3
Asian	6.8
Oceania	0.3
Unknown/Other	13.1
*Birth place (N = 388)*	
Canada	82.2
Other	17.8

SD, standard deviation.

### Use of probiotic products

A large proportion of mothers reported that they had personally used probiotic products (89.3%) (Table 2). The most cited reason for using probiotics was hearing about them via the media or internet (43.3%). One in five mothers (20.3%) used them on recommendation from a healthcare professional (physician, pharmacist or midwife) while a quarter (25.2%) used them following recommendation by a friend.

**Table 2:** **Use of probiotics by mothers and infants and reason for usage**

	Maternal (%)	Infant (%)
Use of probiotics (N = 413)	89.3	50.8
Reason for usage (N = 413)^a^:		
I read/heard about probiotics on TV/internet/newspaper/magazine	43.3	13.6
Recommendation from friend/relative	25.2	10.7
Recommendation from physician/pharmacist	16.2	12.6
Recommendation from naturopath	12.6	5.3
Recommendation from midwife	4.1	1.9
Recommendation from lactation consultant	2.2	0.5
Probiotics have helped me in the past	20.8	4.6
Other	17.2	16.9

^a^Participants were able to select more than one choice or skip the question, therefore the percentages do not add up to 100%.

Half of mothers reported giving a probiotic product to their infant (50.8%). The main reasons indicated for doing so included information from the media, recommendation from a healthcare professional and/or recommendation from a friend or relative (Table 2). Maternal use of probiotics was found to be positively associated with their use in infants (Pearson Chi-Square test P < 0.001; OR 3.99 95%CI:1.75-9.14). There were no significant differences in demographic characteristics (age, marital status, education, income, ethnic origin or birth place) between mothers that reported giving probiotic products to their infants and those that did not.

### Awareness and understanding of probiotics

Awareness of probiotics was high, with 99.3% of mothers reporting that they had heard of the term.

Mothers were subsequently asked to provide their own definition of probiotics (“Please describe what you think probiotics are”). The majority (73.6%) of the 402 respondents who answered this question correctly indicated that probiotics were or contained bacteria, microbes, microorganisms or they used other similar terminology. Only a minority of mothers (4.2%) wrongly described them as other entities such as enzymes, proteins, plant extracts, nuts, herbal supplements or vitamins/minerals.

Below are a selection of typical responses to the question, “Please describe what you think probiotics are.”:

*“Probiotics are micro-organisms found in things like yogurt. They are beneficial and aid in digestion.”*

*“Healthy bacteria for the gut”*

*“Good bacteria that help with the normal functioning of the digestive system”*

*“Bacteria, the good ones”*

*“Product that helps the healthy bacteria in your intestines”*

*“Something good for you that is in some kinds of yogurts”*

**Figure 1:**
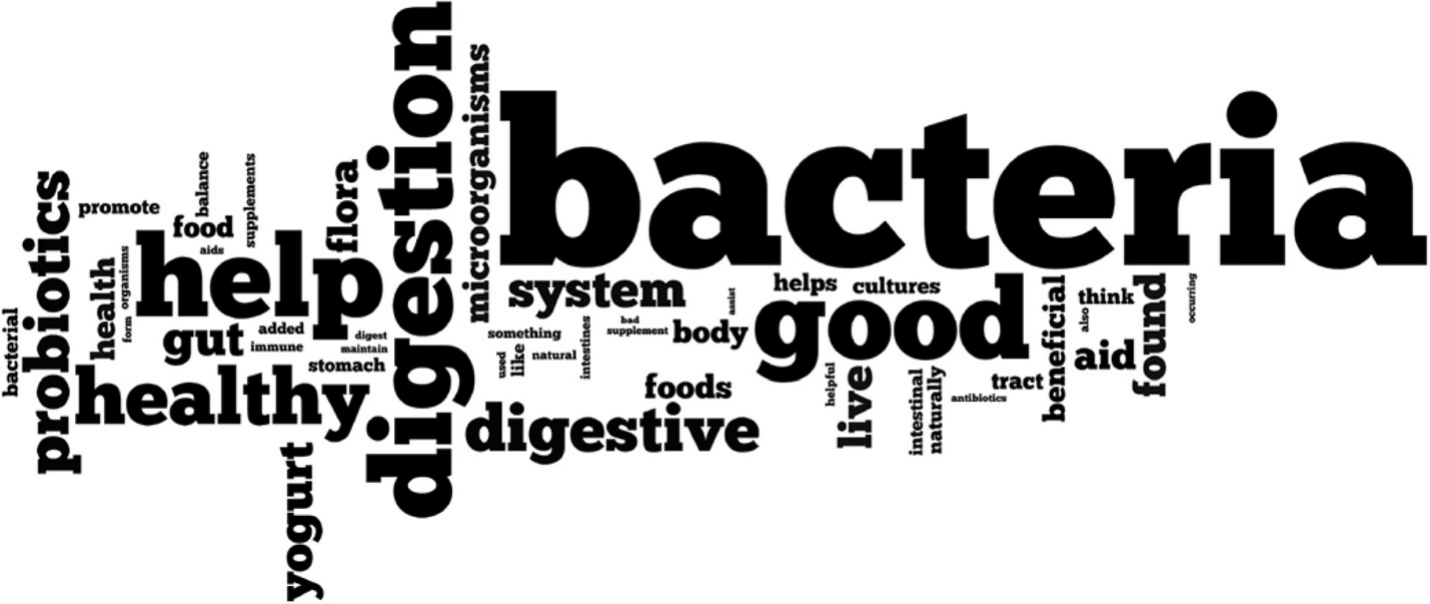
**Word cloud illustrating the top 50 words used to describe probiotics by mothers.** Created using open access Wordle (http://www.wordle.net/). Word size relates to frequency of use.

The majority (88.1%) of mothers used positive words or phrases to describe probiotics such as “good”, “beneficial”, “healthy”, or provided positive health benefits that they associated with probiotic products. None of the mothers’ responses contained negative statements concerning probiotics or their health effects. Although not explicitly asked, many mothers also described health benefits they believed, or had heard, to be associated with probiotics. The most cited were benefits relating to digestion such as “good for digestion”, “gut health”, “maintains GI function” or other similar expressions. Figure 1 illustrates the 50 most frequent words used by mothers to describe probiotics.

Mothers were next asked to provide their opinions on statements about probiotics (Table 3); the vast majority of mothers believed that probiotic products contained live bacteria (87.0%) and that probiotics can alter a person’s intestinal microbiota (82.8%), defined as the bacteria in a person’s digestive system (Table 3).

**Table 3:** **Maternal beliefs of probiotics**

Please indicate your agreement with the following statements N = 407(%)	Agree	Disagree	Don’t know
Probiotics contain live bacteria	87.0	1.7	11.3
Probiotics can alter a person’s microbiota (the bacteria in a person’s digestive system)	82.8	4.2	13.0
The benefit/effectiveness of using probiotics in infants has been confirmed by scientific research	27.3	5.9	66.8

### Benefit and safety of probiotics

**Figure 2:**
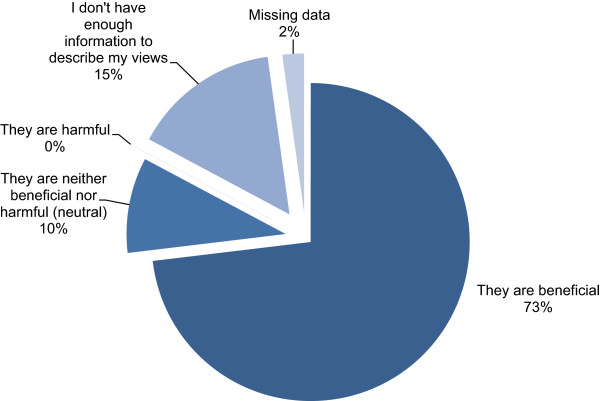
**Maternal view of probiotics as beneficial or harmful.** Mothers were asked to select the statement that best describes their view of probiotic products.

When asked to choose a statement that best described their views of probiotics as harmful or beneficial, almost three quarters (73.1%) of mothers believed that probiotics were beneficial, 9.7% of mothers had neutral views and 15% recorded that they didn’t have enough information to make a decision (Figure 2). No mothers indicated that they thought probiotics were harmful. Mothers who indicated that probiotics were beneficial were more likely to give probiotics to their infants than those who indicated they didn’t have enough information (Pearson Chi-Square test P < 0.001; OR 5.94 95%CI: 3.04-11.63). When asked, over half of the mothers were unsure if the benefit of using probiotics in infants had been confirmed by scientific research (66.8%), while 27.3% agreed and 5.9% disagreed (Table 3). Those agreeing were over twice as likely to give probiotics to their infant as those disagreeing or indicating that they were unsure (P < 0.001; OR 2.42 95%CI: 1.53-3.84).

**Figure 3:**
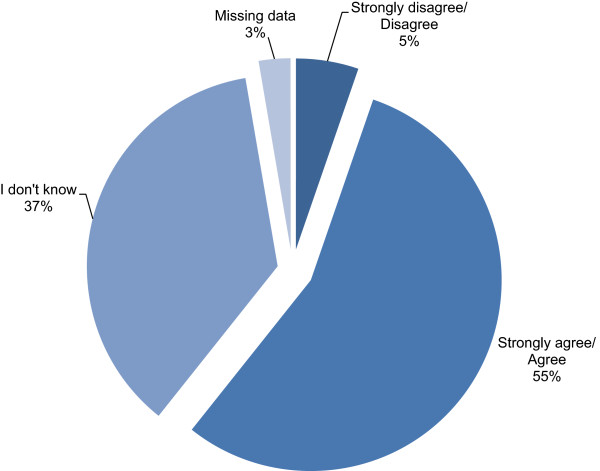
**Maternal agreement with the statement “Probiotics are safe to use for my baby”.**

When asked for their level of agreement with the statement “probiotics are safe to use for my baby”, over half of mothers agreed or strongly agreed that they were safe (55.4%) (Figure 3). Only a minority disagreed or strongly disagreed with the statement (5.3%) while over a third of mothers stated that they were not informed enough to make a decision (36.6%). Mothers agreeing or strongly agreeing to this statement were significantly more likely to give probiotics to their infant than those disagreeing with the statement or indicating that they were unsure (P < 0.001; OR 14.43 95%CI: 8.82-23.59).

In a separate question, mothers were asked to rank how safe they perceived five products to be for infants (organic leafy green vegetables, synthetic multivitamin, antibiotics, homeopathic remedy, probiotics). Results showed that there was a significant difference in perceived safety scores across the five products/foods (non-parametric Friedman Test P < 0.001; Table 4). Inspection of the mean ranks suggests that organic leafy green vegetables were perceived as most safe (mean rank = 1.55) and antibiotics as least safe (mean rank = 4.06). Probiotics were ranked second, after organic vegetables (mean rank = 2.86). Post hoc analysis with Wilcoxon signed-rank tests was conducted with Bonferroni correction applied (significance level set at P < 0.0125) to test for significant difference in perceived safety of probiotics compared to other foods/products. Perceived safety of probiotics was significantly higher than multivitamins, antibiotics and homeopathic remedies (P < 0.001) but lower than organic leafy green vegetables (P < 0.001).

**Table 4:** **Comparison of maternal perception of safety of probiotics and other foods/products for use in infants**

	Frequency (1 = most safe; 5 = least safe) (%)					Missing (%)	Mean Rank ^a^	***P***
	1	2	3	4	5			
Organic leafy green vegetables	80.1	10.7	4.1	1.2	0.7	3.1	1.55	<0.001
Probiotics	18.6	41.6	28.1	6.5	1.5	3.6	2.86	
Synthetic multivitamin	16.9	32.7	30	12.3	4.8	3.1	3.09	
Homeopathic remedy	12.3	28.8	30.8	15.7	8.5	3.9	3.44	
Antibiotics	7.3	17.4	27.8	25.4	19.1	2.9	4.06	

N 392.

SD standard deviation.

^a^Friedman Test.

### Intent to use probiotics based on hypothetical health effects

To assess the potential impact of research findings on mothers’ use of probiotics, we asked them to indicate how likely they would be to give their infant probiotics based on a series of hypothetical research outcomes. Over 80% of mothers indicated that they would definitely or probably give probiotics to their infant if research showed that they reduced the chances of getting a disease (Table 5). If research showed that probiotics have no benefit to health, this number was reduced to 12.8%. When presented with information that health benefits are uncertain and that probiotics may have harmful effects, less than 1.5% would probably or definitely give their infant probiotics, with the vast majority stating that they would definitely not use them (63.2%). When presented with information that giving probiotics to infants permanently changes their microbiota, the majority of mothers would definitely or probably not give probiotics to their infants (59.8%) and only a small minority (9.2%) would definitely or probably administer them. Results showed that mothers were significantly more likely to use probiotics if research showed that these products reduced the chances of their child getting a disease (Wilcoxon signed-rank test; P < 0.001) and significantly less likely to use probiotics if the health effects were uncertain or may be harmful (P < 0.001), and if probiotics were shown to permanently change an infant’s microbiota (P = 0.01) compared to responses if research showed probiotics to have no benefit to health.

**Table 5:** **Maternal intent to use probiotics based on hypothetical research outcomes**

	Likelihood to use probiotics %					
If you had another child, please indicate how likely you would be to give them probiotics if research showed that:	Definitely	Probably	Might	Probably not	Definitely not	***P*** ^***a***^
Giving babies probiotics has no benefit to their health (N = 391)	2.3	10.5	28.6	39.4	19.2	ref
Giving babies probiotics reduces their chances of getting a disease (N = 392)	61.0	26.3	10.5	2.0	0.3	<0.001
The health benefits of giving probiotics to babies is uncertain but may have harmful effects (N = 391)	1.0	0.5	5.9	29.4	63.2	<0.001
Giving probiotics to babies permanently changes their microbiota (i.e. bacteria living in their digestive system) (N = 391)	1.8	7.4	30.9	25.3	34.5	0.01

^a^Wilcoxon signed rank test.

## Discussion

This cross-sectional study of 413 mothers in Alberta, Canada indicates that awareness of probiotics is high, with close to 90% of mothers reporting use of these products. Despite knowing they contain live bacteria, mothers’ attitudes towards probiotics in general were positive and did not elicit any negative or harmful connotations. Many mothers indicated that they had used probiotics themselves and these mothers were four times more likely to have given probiotic products to their infants.

To date, research on attitudes towards probiotics has been limited to studies in adult populations with gastrointestinal problems [16, 17], studies of complementary and alternative medicine use in children [18] and most recently parental perspectives on the use of probiotics in preterm infants [19]. In a cross-sectional study of children attending gastroenterology clinics in Australia, probiotics were used by 50% of children, second only to nutritional supplements (56%) [18]. We report here our finding of similar high usage in our population (51%) of healthy infants and young children.

Since the body of research on the beneficial health effects of probiotics in young children continues to grow, characterizing mothers’ knowledge and opinions on the use of probiotic products is important. Our study showed that mothers rank probiotics as “safer” for infants than other commonly used products such as vitamins, herbal remedies and antibiotics, suggesting that mothers see probiotics as relatively low-risk. The finding is comparable to research on patients with gastrointestinal disorders who similarly viewed probiotics as a more natural and low-risk therapeutic option compared to pharmaceutical treatments [17]. It has been argued that the marketing of probiotics and their widespread availability at grocery stores reinforces this perception among consumers [20]. However, the relatively low use in infants compared to mothers’ usage (51 vs 89%), and maternal uncertainty over their safe use in infants suggests that mothers are more cautious about their use in this population group or unsure of the benefits to their infant. Indeed, many mothers stated that they were uncertain whether probiotic use in infants has been proven to be beneficial by scientific research. Many also indicated that they didn’t have enough information to describe their views of probiotics or safety for use in infants. This result is unsurprising given that current research on benefits of probiotics in prevention and treatment of pediatric disorders is conflicting and in many cases not yet convincing enough to warrant healthcare recommendations.

An interesting finding from our study was that whilst the majority of mothers acknowledged that probiotics can alter a person’s microbiota, when presented with the hypothetical scenario that probiotics could permanently alter their infant’s microbiota, the majority would choose not to use them. While a permanent change could be “permanently beneficial”, this scenario was clearly perceived as negative by mothers in the current survey. In reality, research in both adults and older children suggests that probiotic use only transiently alters gut microbiota profiles [21]; however, it is plausible that exposure to probiotics in infancy during this dynamic phase of intestinal microbiota development could cause more persistent changes. Indeed, there is evidence that other types of early life exposures capable of disrupting the normal development of the microbiota, such as antibiotic use and cesarean section delivery, can have a long lasting impact on the gut microbiota of infants [22–25]. Although our present study was not designed to explore this further, it will be important for other studies to investigate the underlying reasons for these views due to their potential to limit mothers’ uptake if therapeutic use of probiotics becomes more common in the future.

There are a number of limitations inherent to self-administered web-based survey designs that must be considered when interpreting the results. The most important consideration is the introduction of bias due to non-response. Our response rate of 31%, although typical of self-administered survey designs [26], is low and it is possible that survey respondents differ substantially from those that did not respond. For example, individuals already aware of, or using, probiotics may be more likely to respond to the survey, thus biasing results regarding usage and knowledge. We were mindful of the potential for non-response bias and took a number of steps in the design and administration of the survey to maximize response rates that have been shown to be effective in other studies [26–28], including official sponsorship and use of a trusted email contact, non-monetary incentives and reminders. Analysis of demographic characteristics of respondents revealed that they were similar in all respects to the total APrON cohort, except for level of education. This fact, coupled with the tendency of the general APrON cohort to over represent older, more educated mothers, may limit the generalizability of our findings to the wider Canadian population. Further, we were unable to survey fathers, who may have different perspectives. Given fathers’ increasing roles in child care [29], fathers’ perspectives need to be taken into account by health practitioners. Finally, whilst the present study provides an initial overview of mothers’ perspectives of probiotic use in infants, the design did not allow for detailed investigation surrounding attitudes. Future qualitative studies using in-depth interview techniques on a broader spectrum of the population will be important to develop greater understanding of parental use of probiotics in infants and to investigate factors important for effective knowledge translation.

## Conclusions

The present cross-sectional study indicates that awareness and understanding of probiotics are high among a relatively well educated group of mothers in Alberta, Canada. Over half of mothers had given probiotic products to their infants. However, for some mothers, there is still uncertainty regarding the benefit of probiotics and their safety in infants. Further studies on the benefits and safety of probiotic use in healthy infants as well as investment in the translation of research findings to parents are warranted to address these concerns.
